# Supplementary material for: Efficacy and safety of intravenous glucocorticoid therapy for IgG4-related ophthalmic disease
Source: PLoS One. 2023 Apr 19;18(4):e0284442. doi: 10.1371/journal.pone.0284442 (PMC10115269; doi:10.1371/journal.pone.0284442)
Supplement: Attachment — Submitted filename: Response to reviewers_f.docx [file pone.0284442.s001.docx]

5. Review Comments to the Author

Please use the space provided to explain your answers to the questions above. You may also include additional comments for the author, including concerns about dual publication, research ethics, or publication ethics. (Please upload your review as an attachment if it exceeds 20,000 characters)

Reviewer #1: Auhtors should address the following issues:
1) Abstract: Give sample size (n) for the oral steroid group in the “The complete response rate was significantly higher in the IV steroid group (n = 30 eyes) than in the oral steroid group (66.7% vs. 38.7%, p = 0.041)” sentence.

🡪 “(n = 31 eyes)” are added just behind the “in the oral steroid group”.

**Abstract, Line 36:**

The complete response rate was significantly higher in the IV steroid group (n = 30 eyes) than in the oral steroid group (n = 31 eyes) (66.7% vs. 38.7%, p = 0.041).

2) Table-1:
a. Although IQR is explained in table footnotes, there is no ‘IQR’ abbreviation on the table.

🡪 The table footnote explaining IQR is deleted.

b. Comparisons per patient is confusing. For example systemic manifestations are compared on a per patient basis, but ‘Head and neck’ was not?

🡪 Systemic manifestations and serologic features are compared on a per-patient basis. Sorry for confusing mistakes on numbers. We added “If not specified, comparisons were on a per-eye basis.” in table legend.

**Methods, Line 146/162:**

If not specified, comparisons are on a per-eye basis.

3) Table-2: Is the follow-up period comparison based on per patient or per eye?

🡪 Follow-up periods were calculated on a per-eye basis (Except for adverse effects comparisons, most comparisons were on a per-eye basis).

4) Are the Cox models for relapse based on per eye or per patient?

🡪 The Cox models for relapse were on a per-eye basis.

5) If the Cox models are based on per eye, how was the lack of independency between the two eyes from the same patient were accounted for in the models?

🡪 Thank you for the good comment. In consultation with the statistics team of our institute, additional analysis considering inter-eye correlation was performed. The results remained unchanged that IV administration of glucocorticoids was associated with a significant reduction in the relapse of IgG4-ROD.

**Methods, Line 131:**

Multivariate Cox regression analysis, with robust standard errors that accounted for inter-eye correlation, was used to evaluate the independent association of risk factors with relapse of IgG4-ROD.

6) Small KM and Cox analysis table presenting total patients/eyes (whichever used) at risk, total number of events/relapses observed for both groups, and hazard ratio estimates from the Cox models on Figure-2 A and B will be very useful.

🡪 We added tables presenting number of eyes at risk/total number and relapses in both groups. Hazard ratio estimates are presented in figure legends due to the space issue.

**Results, line 195:**

(A) In all patients (hazard ratio 5.5; 95% confidence interval 2.4–12.8; p < 0.001). (B) In the subgroups with initial serum IgG4 level > 1.35 g/L (hazard ratio 19.2; 95% confidence interval 6.1–60.9; p < 0.001).

Reviewer #2: The authors applied IV glucocorticoid therapy which is for moderate-to-severe TAO for IgG4-ROD.
As the cumulative dose of steroid was significantly higher in the IV steroid group than in the oral steroid group, such important message should be emphasized in the paper as well as the title.

🡪 Thank you for the good comment. In the abstract, the treatment regimen of oral or IV methylprednisolone was additionally described in detail.

**Abstract, line 29~31:**

Glucocorticoids were orally or intravenously administered, according to the date of treatment. 🡪 Glucocorticoids were given either oral prednisolone at an initial dose of 0.6 mg/kg/day for four weeks with subsequent tapering or once weekly IV methylprednisolone (500 mg for six weeks, then 250 mg for six weeks), according to the date of treatment.
